# Comparison of Two Toric IOLs with Different Haptic Design: Optical Quality after 1 Year

**DOI:** 10.1155/2018/4064369

**Published:** 2018-02-11

**Authors:** Kata Miháltz, Michael Lasta, Michael Burgmüller, Pia Veronika Vécsei-Marlovits, Birgit Weingessel

**Affiliations:** ^1^Department of Ophthalmology, Hospital Hietzing, Vienna, Austria; ^2^Karl Landsteiner Institute of Process Optimization and QM in Cataract Surgery, Vienna, Austria

## Abstract

**Background:**

The purpose of this prospective, randomised study was to interocularly compare the visual performance after implantation of two different toric IOLs with different haptic design.

**Methods:**

59 subjects with corneal astigmatism greater than 1.25 diopter (D) were implanted with an AT TORBI 709M IOL (Carl Zeiss Meditec AG) in one eye and with a Tecnis toric aspheric IOL (Abbot Medical Optics) in the other eye. Observation procedure was performed 12 months postoperatively. Main outcome measures included uncorrected distance visual acuity (UDVA), manifest refraction, IOL rotation, and IOL position.

**Results:**

Mean UCDVA was 0.04 ± 0.14 logMAR for AT TORBI eyes and 0.06 ± 0.15 logMAR for Tecnis eyes (*p* = 0.3). The postoperative spherical equivalent values were significantly lower in the AT TORBI group. Mean toric IOL axis rotation was 3.0 ± 2.26 degrees for AT TORBI eyes and 3.27 ± 2.37 for Tecnis eyes (*p* = 0.5). The mean vertical IOL tilt and vertical decentration values measured with the Visante OCT were significantly larger in the AT TORBI group (*p* < 0.05).

**Conclusions:**

Both the Tecnis and the AT TORBI toric IOLs successfully reduced ocular astigmatism. Emmetropia could be better achieved with the AT TORBI IOL, whereas the Tecnis showed better positional stability. This trial is registered with ICMJE NCT03371576.

## 1. Introduction

Toric intraocular lenses (IOLs) were developed to correct the preexisting astigmatism induced by corneal toricity after cataract surgery. The goal is to provide complete visual rehabilitation for distance vision [1]. New designs of these IOLs with different materials, morphology, haptic design, and calculation algorithms were developed to provide better performance [1–5]. The position and the stability of the IOL in the capsular bag are an important issue as IOL rotation, displacement, or tilt can lead to incorrect optical results and patient dissatisfaction. The purpose of the present study was to compare the effect of different haptic systems on IOL positioning and visual quality. In order to achieve our goal, novel techniques for the assessment of the exact position of the lens with anterior segment OCT and wavefront aberrometry were used.

Several reports have proven the interocular symmetry of biometric parameters like axial length, anterior chamber depth, and lens thickness between right and left eye, which all play an important role in the postoperative refractive outcome [6, 7]. In our study, one IOL was implanted in one eye and the other IOL was implanted in the fellow eye in the same patient, thus allowing a better evaluation of the effect of IOL properties on the optical results by minimalizing the individual anatomical variances. To our knowledge, this is the first article where toric IOLs have been interocularly compared.

## 2. Patients and Methods

### 2.1. Patients and Implants

In this prospective, randomized study, 118 eyes of 59 patients with age-related cataract and corneal astigmatism over 1.25 D on both eyes were included. According to a previous sample size calculations, the study had 80% of power using a paired *t*-test with a two-sided significance level of 0.05. The sample was powered to detect the difference in the rotational stability of the two IOLs.

Each patient received, according to a randomization list, based on random permutation numbers one of the two lenses in one, and the second lens in the other eye. One of the lenses was the Technis toric aspheric IOL (Abbot Medical Optics Inc., Santa Ana, CA, USA), which is a one-piece, loop-haptic, aspheric hydrophobic acrylic intraocular lens with three-point fixation and an anterior offset haptics. The other lens is the AT TORBI 709MP (Carl Zeiss Meditec AG, Jena, Germany), which is a plate-haptic, bitoric, aspheric hydrophilic acrylic IOL. The Tecnis toric IOL aims a full correction of mean spherical aberration (SA: −0.27 *μ*m), whereas the AT TORBI is a SA neutral IOL (SA: 0.0 *μ*m).

Each patient has undergone a complete ophthalmological evaluation. Subjects with previous ocular surgery, trauma, ocular disease other as cataract, poorly dilated pupils, or known zonular weakness were excluded from the study.

The study was conducted in compliance with the Declaration of Helsinki, the Good Clinical Practice (ICH + GCP) of the WHO, as well as with applicable country and local requirements regarding ethics committee/institutional review boards, informed consent, and other statutes or regulations regarding protection of the rights and welfare of human subjects participating in biomedical research.

### 2.2. Surgery

Five experienced surgeons performed all cataract extractions under local anaesthesia. Preoperative, marking of the horizontal axis in seated position was performed. The self-sealing 2.4 mm superior-temporal incision (at 120 degree), injection of viscoelastic substance, capsulorhexis (with 360° overlapping edges), phacoemulsification, irrigation/aspiration of cortical material, and injection of viscoelastic substance into the capsular bag were performed as standard procedures. The IOL was implanted via injector into the capsular bag followed by thorough aspiration of the viscoelastic substance from the eye. The IOL axis was positioned on the planned meridian using a Mendez Marker Ring.

### 2.3. Preoperative and Postoperative Examination

Preoperatively, all patients had a complete ophthalmic examination. Biometry using the IOL Master (Carl Zeiss Meditec AG) was performed. The ULIB optimized IOL constant was used to determine IOL power for the Tecnis lens (A). The IOL power corresponding to the smallest minus refractive error, based on the IOL Master measurements, was chosen. The IOL cylinder power of the Tecnis lens and both the spherical and cylindrical power of the TORBI lens were calculated using the online Toric Calculators provided for each lens by its manufacturer, based on anterior corneal astigmatism values. Follow-up assessments were performed 1 week and 1, 3, 6, and 12 months postoperatively.

### 2.4. Assessment of IOL Position

Axial alignment of the toric IOL was measured using the tilted narrow light of the slit lamp (Haag-Streit, Bern, Switzerland), which was adjusted to the marking on the IOL, and the degree of axial alignment was read by the examiner.

IOL tilt and decentration were measured with the Visante omni anterior segment OCT (Carl Zeiss Meditec AG) as described by Kumar et al. [8]. Postoperative anterior chamber depth (ACD) was also measured with the Visante, between the anterior corneal surface and the anterior IOL surface.

### 2.5. Aberrometry Measurements

Total, corneal, and internal optical aberrations were measured using the iTrace VFA Visual Function Analyser (Tracey Technologies, Houston, TX). This device measures keratometry, autorefraction, pupil diameter, topography, and wavefront aberrations simultaneously on the same axis [9, 10]. Images were recorded with the patient focusing on a distant target with dilated pupils and a fixed entrance pupil scan size of 4.0 mm. Visual quality was described by RMS (root mean square), HORMS (higher-order root mean square), MTF (modulation transfer function), and Strehl ratio. The average height of the MTF curve for higher-order aberrations was selected for further analysis.

### 2.6. Data and Statistical Analyses

Pre- and postoperative cylindrical refractive errors were expressed as power vectors: as Jackson crossed cylinder J0 with axes at 90 degrees and 180 degrees and as J45 with axes at 45 degrees and 135 degrees as described by Thibos and Horner [11].

Statistical analyses were performed with SPSS 19.0 (SPSS Inc., Chicago, IL, USA). Univariable and multivariable regression analyses via generalized estimating equation (GEE) models were used to compare visual, refractive, aberrometric, and IOL alignment parameters between the study groups. In GEE models, data from the two eyes of the same subject were statistically analysed as repeated measures. Thus, this analysis takes into account the correlated nature of data from the two eyes of the same patient and provides valid *p* values for group comparisons [12].

## 3. Results

The study enrolled a total of 118 eyes of 59 patients with age-related cataract and corneal astigmatisms over 1.25 D. Mean age was 66.83 ± 11.54 years in the study group, and the female/male ratio was 33/26.  Table 1 shows the patients' preoperative data. There were no intraoperative complications. 2 eyes required repositioning of the IOL postoperatively in the AT TORBI group while 4 eyes in the Tecnis group, because of misalignment greater than 10°. Each repositioning occurred during the first postoperative month.

### 3.1. Visual Acuity

Table 2 shows the postoperative refractive and visual acuity results in the two study groups. At the 12-month visit, no significant difference was found in uncorrected (UDVA) and corrected (CDVA) distance visual acuity between the groups. The UDVA was 0.1 logMAR or better in 88% of the eyes with the AT TORBI lens and 84% of the eyes with the Tecnis IOL. The CDVA was 0.1 logMAR or better in 98% of the eyes with the AT TORBI lens and 96% of the eyes with the Tecnis IOL.

### 3.2. Refraction

**Figure 1:**
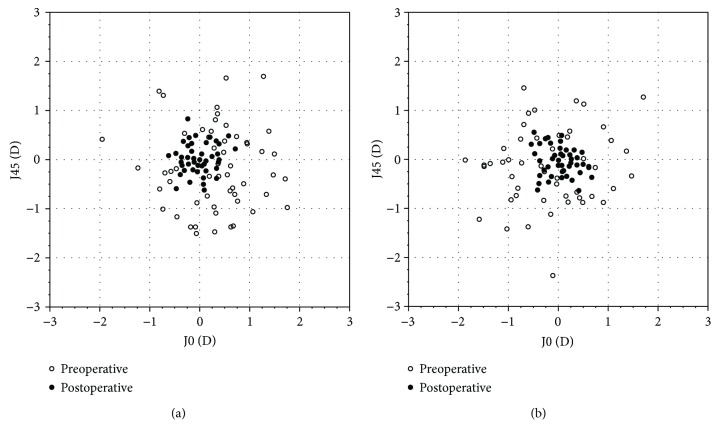
The distribution of preoperative and postoperative refractive cylinder in the 2 groups. (a) AT TORBI. (b) Tecnis.

Twelve months postoperatively there was no significant difference in manifest refractive cylinder between the two groups (Table 2).  Figure 1 shows the preoperative and postoperative J0 and J45 refractive cylinder vectors in the two groups; it is visible that postoperatively the data are more concentrated around the origin. However, there is no difference in the postoperative reduction of the magnitude of the refractive J0 and J45 vector between the two lenses (Table 3). The postoperative manifest refractive sphere and spherical equivalent (SE) values were significantly lower in the AT TORBI group than in the Tecnis group, yet there was no statistically significant difference in the mean predicted spherical error between the two IOLs (Table 2). The change between pre- and postoperative sphere and SE values showed no difference in the two groups as seen in Table 3. 58% of the eyes obtained a postoperative spherical equivalent within ±0.5 D in the AT TORBI group and 62% in the Tecnis group.

### 3.3. Aberrations and Visual Quality

The postoperative *whole eye* aberrations and image quality characteristics of the two groups are presented in Table 4. The difference in total ocular RMS and HORMS between the two study groups was not significant. There was no clinically significant difference in the corneal abberations between the two study groups (*p* > 0.05). The ocular vertical tilt (Z_1_^−1^) was significantly lower in the Tecnis group whereas the ocular horizontal tilt (Z_1_ [1]) was similar in the two groups. The ocular vertical coma (Z_3_^−1^) was significantly lower in the Tecnis group as well, but not the ocular horizontal coma (Z_3_ [1]). The Strehl ratio for all aberrations showed no difference between the groups; however, the Strehl ratio for higher-order aberrations was barely significantly higher in the Tecnis group (*p* = 0.07). Due to the different asphericity of the two IOLs, the spherical aberration (Z_4_^0^) of the AT TORBI group was significantly larger. The average height of the ocular MTF curves for higher-order aberrations was significantly higher in the Tecnis group than in the AT TORBI group, whereas the MTF for all aberrations did not differ.

### 3.4. IOL Alignment

The IOL rotation measured at the 12th month follow-up visit was not statistically different between the two study groups (Table 4). The mean postoperative rotation measured on the slit lamp was 3.0 ± 2.26 degrees in the AT TORBI group and 3.27 ± 2.37 degrees in the Technis group. Table 4 also shows that only a small amount of IOL rotation could be observed after the first postoperative week: 0.18 ± 2.04 degrees in the AT TORBI group and 0.36 ± 2.16 degrees in the Tecnis group.

**Figure 2:**
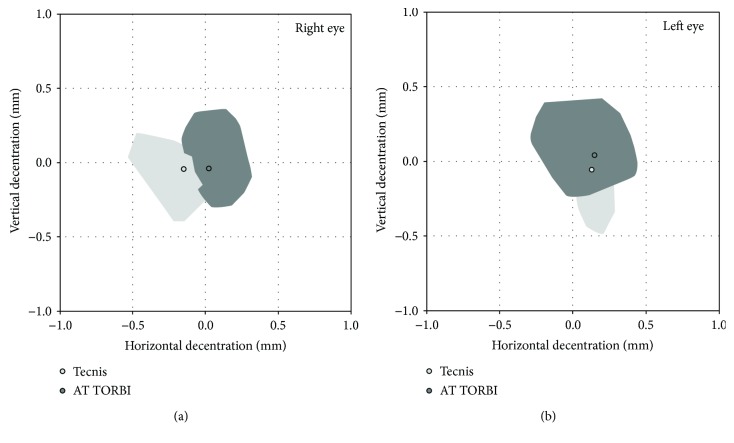
Mean values of horizontal and vertical decentration in the two study groups, separately depicted for right and left eyes.

The mean vertical IOL tilt and vertical decentration values measured with the Visante OCT were significantly larger in the AT TORBI group. Horizontal tilt and decentration showed no difference between the two groups.  Figure 2 shows the mean values of horizontal and vertical decentration in the two study groups, separately depicted for right and left eyes.

**Figure 3:**
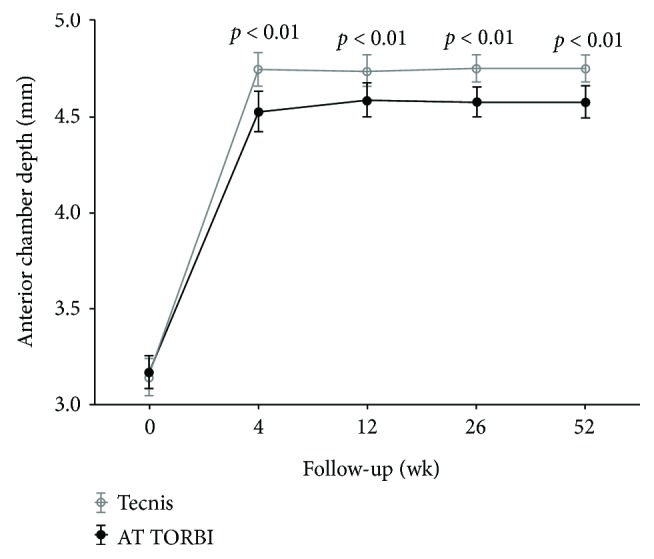
Distribution of anterior chamber depth mean values measured with the Visante OCT preoperatively and 1, 3, 6, and 12 months postoperatively in the two study groups. Error bars indicate standard deviation.

**Figure 4:**
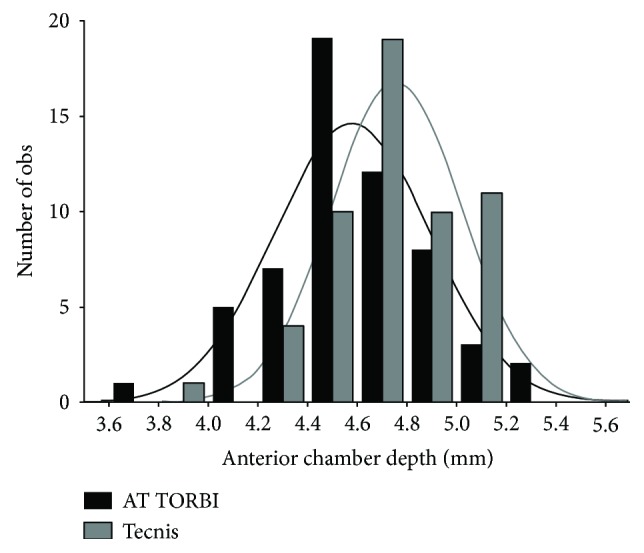
Distribution of postoperative ACD values (mm) 12 months postoperatively in the two study groups.

Postoperative anterior chamber depth values measured with the Visante OCT at the first, third, sixth, and twelfth month visit were all significantly (*p* < 0.001) deeper in the Tecnis group (mean ACD value at month 12: 4.75 ± 0.26 mm) as in the AT TORBI group (mean ACD value at month 12: 4.58 ± 0.31).  Figure 3 shows the mean values of pre- and postoperative anterior chamber depth in the two study groups. No further significant deepening or flattening of the anterior chamber could be observed after the first postoperative month in none of the groups. The histogram on Figure 4 shows the distribution of postoperative ACD values (mm) 12 months postoperatively in the two study groups.

## 4. Discussion

The purpose of this study was to evaluate the postoperative lens position stability and its effect on refractive and optical quality in eyes with two different toric IOLs. Several studies have evaluated the effectiveness of the Tecnis toric [2, 3, 13, 14] and the AT TORBI [5, 15, 16] IOLs to correct corneal astigmatism, but to our knowledge, this is the first one, which compares them intraindividually. The advantage of a plate haptic design versus open loop haptic design of toric IOLs is still a question of debate. Patel et al. [17] found that plate haptic IOLs show greater rotational stability than loop haptics, anyhow a more recent study of Scialdone et al. [15] could not confirm this finding. In this study, the difference in mean IOL misalignment 12 months after implantation was not significant. The majority of the rotations occurred during the first postoperative week, while the capsular bag is still open and remains of viscoelastic material might be present. This fact confirms the theory that the rotating effect of late constrictive forces due to capsular fibrosis and shrinkage can be minimized through symmetric in-the-bag fixation [17].

The two IOLs have also shown similar visual results 12 months after implantation. The analysis of postoperative refractive data indicated that eyes with the AT TORBI lens were closer to emmetropia, than eyes with the Tecnis. Regarding postoperative cylindrical refractive error, no difference could be observed between the two groups. The explanation for the mild myopic shift observed in the Tecnis group may lie in the different power computation methods of the two IOLs. Whereas the Tecnis toric calculator requires an IOL spherical value chosen by the surgeon and calculated with a standard IOL formula, the Zeiss program uses raw biometric data (keratometry data, axial length) and also necessitates the preoperative anterior chamber depth value and estimates the postoperative ACD. Another difference between the two methods is that the Zeiss algorithm uses meridional analysis for calculating the spherocylindrical IOL power. This means that IOL power is calculated separately for both the steep and flat corneal meridians instead of using the mean keratometry value. According to Fam and Lim [18], this method allows a precise prediction of the expected spherocylindrical refraction. As the algorithm of the Zeiss calculator has not been published, we cannot further analyse the differences between the principles of the two systems.

Several studies have addressed the importance of estimated effective lens position (ELP) on IOL power prediction accuracy, which is also influenced by the haptic design [19–21]. The anteriorly offset haptics of the Tecnis lens enable a three-point fixation with the posterior capsule. According to Miyata et al. [22], the one-piece Tecnis IOL showed a better stability than the three-piece IOL. They measured a mean postoperative ACD of 4.14 mm in eyes with mean AL of 23.37 mm twelve months after surgery with Scheimpflug camera, not including the corneal thickness. In another paper, Weber et al. [20] found that the mean postoperative ACD value of the same IOL was 4.81 mm. In our study, the mean ACD value was 4.75 ± 0.26 mm at month 12 and did not significantly change after the first postoperative month.

Other than the Tecnis IOL with its anterior offset haptics, the plate haptic TORBI lens with its broad haptic-optic junction does not bend the posterior capsule. This explains our finding that the ACD in the Tecnis group was significantly deeper as in the AT TORBI group, mean value of this was 4.58 ± 0.31 mm. In a similar setting, Hirnschall et al. [23] measured a mean postoperative ACD value of 4.53 mm for the same lens. The slight backward shift they observed during the first postoperative month could not be confirmed by us.

Further analysing the distribution of postoperative ACD values of the two IOLs (Figure 4), the deeper position of the Tecnis IOL is not the only visible difference. The Gaussian curve of the Tecnis IOL is also higher and narrower, suggesting a more predictable postoperative position of the IOL, although the anatomical conformation of the two eyes of the same person is mostly similar. This might be the reason why the vertical tilt and decentration were smaller in this later group, where gravitation forces are more influent as in the case of horizontal tilt and decentration. The postoperative aberrometric findings also confirm this theory, as far as the Tecnis group showed higher values of vertical tilt (Z_1_^−1^) and coma (Z_3_^−1^). The Strehl ratio and the height of the modulation function (MTF) curve for higher-order aberrations were both larger in the Tecnis group, indicating a better optical quality. Due to the different asphericity of the two IOLs, the spherical aberration (Z_4_^0^) of the AT TORBI group was significantly greater. Aberrations is correcting aspheric IOLs as the Tecnis aim a full correction of the mean corneal spherical aberration (SA), which leads to an improved contrast sensitivity and optical quality. As corneal asphericity was similar in the groups, the better Strehl ratio and MTF values are probably due to the IOL.

Our study has confirmed the previous findings that both lenses have a high degree of rotational and centration stability while the refractive outcome was better in the AT TORBI group and the SA correction was better in the Tecnis group.

There are some limitations of this study. The IOL constants of the formulas calculating the spherical power of the Tecnis lens were not personally optimized, which would avoid systematic errors in the preoperative measurements, instead they were taken from the ULIB database [24]. Furthermore, the IOL calculation was performed with two different platforms, which is a limitation by the comparison. Nevertheless, according to a recent study of Weber et al. [20], the optimized A constant of the Tecnis IOL was the same as the one given in the ULIB database.

## Figures and Tables

**Table 1:** Preoperative characteristics of the two study groups.

	AT TORBI IOL (*n* = 59)		Tecnis toric IOL (*n* = 59)		*p*
	Mean ± SD	Range	Mean ± SD	Range	
CDVA (logMAR)	0.32 ± 0.21	0–1	0.33 ± 0.18	0–1	0.81
Spherical error (D)	−1.81 ± 3.75	−13.75–5.00	−1.62 ± 3.63	−14.75–6.00	0.79
Cylindrical error (D)	2.13 ± 0.91	0.50–4.25	2.1 ± 0.93	0.50–4.75	0.67
Axis (degree)	94.97 ± 42.16	0–180	86.71 ± 42.86	5–175	0.31
SE (D)	−0.71 ± 3.51	−12.25–5.87	−0.55 ± 3.43	−12.87–7.00	0.83
J0 (D)	0.28 ± 0.74	−1.96–1.76	−0.12 ± 0.73	−1.88–1.70	0.07
J45 (D)	−0.15 ± 0.80	−1.50–1.70	−0.13 ± 0.73	−2.36–1.47	0.84
Corneal cylinder (D)	2.06 ± 0.64	1.00–3.96	2.02 ± 0.61	1.26–3.95	0.68
Corneal SA (*μ*m)	0.31 ± 0.38	0.01–2.84	0.27 ± 0.17	0.04–1.20	0.48
Axial length (mm)	23.29 ± 1.19	21.13–26.82	23.31 ± 1.29	21.01–27.33	0.94
Spherical IOL power (D)	19.84 ± 3.66	11.5–28	20.88 ± 3.85	11.0–32.0	0.001
Cylindrical IOL power (D)	2.41 ± 0.88	1.0–5.0	2.52 ± 0.74	1.5–4.0	0.68
CCP (D)	44.27 ± 1.5	41.2–47.8	44.2 ± 1.5	40.9–48.1	0.9

CDVA = corrected distance visual acuity; logMAR = logarithm of the minimum angle of resolution; SE = spherical equivalent; J0 and J45 = cylindrical vectors; SA = spherical aberration (central 6 mm); CCP = central corneal power; P = difference between the two groups using generalized estimating equation.

**Table 2:** Postoperative results in the two study groups.

	AT TORBI IOL		Tecnis toric IOL		*p*
	Mean ± SD	Range	Mean ± SD	Range	
UDVA (logMAR)	0.03 ± 0.13	−0.2–0.5	0.05 ± 0.14	−0.2–0.6	0.31
CDVA (logMAR)	−0.04 ± 0.09	−0.2–0.4	−0.03 ± 0.12	−0.2–0.3	0.35
Spherical error (D)	−0.51 ± 0.58	−1.5–1.0	−0.75 ± 0.51	−1.75–0.5	0.014
Spherical PE (D)	0.49 ± 0.59	−0.57–1.52	0.43 ± 0.62	−0.43–1.62	0.76
Cylindric error (D)	0.68 ± 0.41	0–1.75	0.74 ± 0.38	0–1.50	0.45
SE (D)	−0.15 ± 0.61	−1.5–1.43	−0.36 ± 0.51	−1.5–0.62	0.02
J0 (D)	−0.03 ± 0.27	−0.62–0.71	0.02 ± 0.29	−0.54–0.66	0.35
J45 (D)	0.004 ± 0.29	−0.62–0.84	−0.018 ± 0.29	−0.63–0.56	0.67
Corneal SA (*μ*m)	0.44 ± 0.25	0.12–1.08	0.41 ± 0.25	−0.60–1.15	0.83
CCP (D)	44.23 ± 1.7	40.5–47.8	44.16 ± 1.7	40.9–47.8	0.83

CDVA = corrected distance visual acuity; UDVA = uncorrected distance visual acuity; logMAR = logarithm of the minimum angle of resolution; PE = predicted error; J0 and J45 = cylindrical vectors; SE = spherical equivalent; SA = spherical aberration (central 6 mm); CCP = central corneal power; dRotation (1 w–12 m) = difference of IOL rotation between the first week and 12th month; dAP (SL-iTrace) = difference of the measured axis placement with the two methods: SL = slit lamp and with the iTrace; P = difference between the two groups using generalized estimating equation.

**Table 3:** Changes from the baseline to the month 12 visit.

Changes	AT TORBI IOL		Tecnis toric IOL		*p*
	Mean ± SD	Range	Mean ± SD	Range	
Spherical error (D)	2.39 ± 2.73	−1.25–13.00	2.15 ± 2.59	−1.25–14.00	0.61
SE (D)	2.18 ± 2.46	−1.12–11.62	2.02 ± 2.35	−1.00–12.50	0.73
J0 (D)	0.43 ± 0.50	−0.50–1.95	0.47 ± 0.52	−0.37–1.72	0.64
J45 (D)	0.45 ± 0.48	−0.36–1.45	0.34 ± 0.50	−0.50–2.25	0.20
SIA (D)	0.45 ± 0.25	0.12–1.08	0.44 ± 0.31	0.06–1.65	0.92
Corneal SA (um)	0.03 ± 0.18	−0.30–0.90	0.03 ± 0.62	−0.65–1.37	0.97

SE = spherical equivalent; SA = spherical aberration (central 6 mm); J0 and J45 = cylindrical vectors; SIA = corneal plane surgically induced astigmatism; P = difference between the two groups using generalized estimating equation.

**Table 4:** Postoperative *ocular* aberrations, visual quality, rotation, tilt, and decentration in the two study groups.

	AT TORBI IOL	Tecnis toric IOL	*p*
	Mean ± SD	Mean ± SD	
RMS (*μ*m)	0.60 ± 0.34	0.61 ± 0.33	0.96
Tilt vertical (*μ*m)	0.13 ± 0.32	0.03 ± 0.35	0.05
Tilt horizontal (*μ*m)	−0.01 ± 0.24	−0.01 ± 0.31	0.93
Strehl	0.05 ± 0.04	0.05 ± 0.04	0.74
MTF	0.26 ± 0.10	0.25 ± 0.08	0.74
*Higher-order terms*			
HORMS (*μ*m)	0.26 ± 0.15	0.27 ± 0.18	0.74
Coma vertical (*μ*m)	0.04 ± 0.09	0.01 ± 0.09	0.04
Coma horizontal (*μ*m)	−0.01 ± 0.07	−0.006 ± 0.07	0.68
SA (*μ*m)	0.05 ± 0.06	0.01 ± 0.04	<0.001
HO Strehl	0.11 ± 0.08	0.13 ± 0.09	0.07
HO MTF	0.35 ± 0.11	0.39 ± 0.12	0.04
Rotation SL (deg)	3.0 ± 2.26	3.27 ± 2.37	0.55
Vertical tilt (mm)	0.46 ± 1.38	−0.29 ± 1.68	0.013
Horizontal tilt (mm)	0.84 ± 1.47	0.65 ± 1.64	0.48
Ver. decentration (mm)	0.02 ± 0.21	−0.06 ± 0.20	0.04
Hor. decentration (mm)	0.07 ± 0.34	−0.06 ± 0.04	0.07

RMS = root mean square; HORMS = higher-order root mean square; MTF = modulation transfer function; SA = spherical aberration; P = difference between the two groups using generalized estimating equation; Rotation SL = rotation measured at the slit lamp.
